# Supplementary material for: Ensemble forecast of human West Nile virus cases and mosquito infection rates
Source: Nat Commun. 2017 Feb 24;8:14592. doi: 10.1038/ncomms14592 (PMC5333106; doi:10.1038/ncomms14592)
Supplement: Supplementary Information — Supplementary figures, supplementary tables, supplementary methods and supplementary references. [file ncomms14592-s1.pdf]

Supplementary Information

Supplementary Methods

The forecast system relies on three components: 1) a core compartmental epidemiological model that can freely simulate the spread of West Nile virus (WNV) in mosquitoes, birds, and humans; 2) WNV surveillance data (i.e., vector mosquito WNV infection rates and reported human WNV cases); and 3) a data assimilation method (here the ensemble adjustment Kalman filter [EAKF]). The data assimilation method uses the surveillance data to recursively inform and optimize an ensemble of model simulations and in so doing provide an improved, posterior estimate of the true state, as well as unobserved state variables and parameters. Forecasting is then generated in two successive steps. First, an ensemble of model simulations is iteratively optimized using the EAKF and weekly observations of estimated mosquito infection rates and human WNV cases for a particular season until the week at which a forecast is to be initiated (in real time, this would be the current week). Through the recursive EAKF optimization, model variables and parameters are better aligned with the local dynamics of the outbreak as thus far observed. Next, an ensemble forecast is generated by integrating the optimized ensemble of model simulations through to the end of the season. The remainder of this section further details development of the SIR-compartmental model, the observational data, and data assimilation methods (i.e., EAKF), as well as the forecast procedure and system validation.

WNV Model Development. WNV is maintained in an enzootic cycle in which vector mosquitoes interact with avian hosts[6]. Higher proportions of infected mosquitoes increase the chance of human contact with an infected mosquito and risk of spillover transmission to humans. We developed a mathematical model to depict both the enzootic WNV transmission cycle and spillover transmission to humans.

The model is a compartmental structure representing the WNV infection status of mosquitoes, birds and humans. The model uses a standard susceptible-infected-recovered (SIR) epidemiological construct in which all compartments are perfectly mixed. Population numbers are assumed constant for the mosquitoes, birds and humans during each outbreak; for the mosquitoes birth equals death; for the birds and humans no birth or death was simulated.

The basic model structure is as follows:

$$\frac{dS_M}{dt} = \mu_M N_M - \beta S_M \frac{I_B}{N_B} - \mu_M S_M - \alpha S_M \qquad \textbf{[S1]}$$

$$\frac{dI_M}{dt} = \beta S_M \frac{I_B}{N_B} - \mu_M I_M + \alpha S_M \qquad \textbf{[S2]}$$

$$\frac{dS_B}{dt} = -\beta \frac{S_B}{N_B} I_M \qquad \textbf{[S3]}$$

$$\frac{dI_B}{dt} = \beta \frac{S_B}{N_B} I_M - \frac{I_B}{\delta_B} \qquad \textbf{[S4]}$$

$$\frac{dI_H}{dt} = Poisson(\eta I_M) \qquad \textbf{[S5]}$$

where S_M is the number of susceptible mosquitoes, μ_M is the mosquito birth and death rate, N_M is the mosquito population, t is time in days, β is the contact rate, or probability of transmission between birds and mosquitoes, α is the rate of WNV seeding prior to day 200, I_M is the number of infected mosquitoes, N_B is the bird population, I_B is the number of infected birds, S_B is the number of susceptible birds in the population, δ_B is the recovery rate of birds, I_H is the number of infected humans, and η is a scaling factor representing the probability of spillover transmission to humans from mosquitoes. WNV spilling over to humans is simulated by a Poisson random number generator to account for the uncertainty pertaining to few chance human cases and the start of a large-scale outbreak.

Study Area. Suffolk County was one of the first places in the western hemisphere to experience and identify WNV. Since the virus was first discovered in 1999, it has annually infected a combination of birds, horses, humans, and mosquitoes. Suffolk County is a suburban county with a population of approximately 1.5 million people located east of New York City. The county occupies the central and eastern parts of Long Island, NY with a land area of 912 square miles. The western portion of the county is predominantly densely populated residential and commercial properties and the east contains more open spaces and agriculture. Natural woodlands and freshwater wetlands are found throughout the county and salt marshes are primarily on the south shore and east end. The natural landscape and land use patterns throughout the county provide ample breading sites for *Culex spp*. mosquitoes, the primary vectors of WNV.

Observed Human Cases. Weekly human cases of WNV in Suffolk County New York were obtained from ArboNET, the national arboviral surveillance system, from 2001 to 2014[30]. The CDC and State health departments developed ArboNET, an electronic surveillance system, in 2000 to monitor the emergence of WNV in the western hemisphere. WNV is classified as a nationally notifiable disease, requiring state and local health departments to report the weekly number of human WNV cases to the Centers for Disease Control and Prevention through the ArboNET surveillance system[31].

Weekly reported human cases of WNV, both neuroinvasive and non-neuroinvasive, were used in this study. Human cases of WNV were aggregated by week according to the date of illness onset with each week defined as Sunday to Saturday. Supplementary Fig.1 shows the weekly variability among years. We assumed the error variance associated with each weekly observation, i.e. the observational error variance (OEV) was half the reported number of WNV cases for that week. If zero or one case was reported, then the OEV was set to one.

Observed Infectious Mosquitoes. Weekly *Culex spp*. mosquito WNV infection rates were estimated from Suffolk County surveillance data from 2001 to 2014. The Suffolk County Department of Health Services, Arthropod-Borne Disease Laboratory, conducted weekly mosquito surveillance each year from early June to the middle of October. At the beginning of each season, trap locations were optimally set to survey the whole county while integrating information on the historical presence of WNV. As the season progressed, mosquito surveillance was expanded within regions where mosquitoes tested

WNV positive and to regions where WNV had been identified in birds, horses or humans. Total traps within a season ranged from 47 to 104 and on average 29.3 (SD=13.8) traps were set weekly.

Mosquito collections of both gravid and host-seeking mosquitoes were made weekly using the Centers for Disease Control and Prevention (CDC) gravid and CDC light traps (John W. Hock Co., Gainesville, FL), respectively. Gravid traps were baited with rabbit-chow infusion and light traps were baited with dry ice. Trapped mosquitoes were identified and separated by species. Only *Culex spp*. mosquitoes were considered in this analysis.

Our study focuses on WNV-assayed pools of *Culex* spp. mosquitoes as only a few *Culex* mosquito species drive enzootic transmission[6]. In the northeast U.S., the mosquito-bird transmission cycle is thought to occur primarily via *Cx. pipiens* and *Cx. restuans*[36,37] and the risk of human spillover is primarily due to *Cx. pipiens*[38,39] and *Cx. salinarius*[36,37]. All three species of Culex mosquitoes are found in Suffolk County. *Cx. pipiens, Cx. restuans,* and *Cx. salinarius* have similar morphological characteristics making it difficult to distinguish them especially when physically damaged during collection[40]. Historically, New York combines *Cx. pipiens* and *Cx. restuans* for arboviral testing, but *Cx. salinarius* may also be unintentionally included in the arboviral analysis[41].

For arboviral analysis, mosquito samples were submitted to the New York State Department of Health (Arbovirus Laboratory, Wadsworth Center) in compliance with state protocol [41,42]. The pooled mosquito samples were analyzed using real-time reverse transcription-polymerase chain reaction (PCR) to determine if a pool (size range 1- 81; mean 33.7; median 31) had at least one WNV positive mosquito[41,42]. All pools over a given week (Sunday to Saturday) were combined to calculated the weekly county-wide mosquito infection rate using a maximum likelihood estimate (MLE)[29].

The *Culex spp*. mosquito data set included 13,346 pools of mosquitoes sampled over 263 weeks. If the total number of mosquitoes sampled within a given week was less than 300 mosquitoes, we averaged the current week's estimate of infectious mosquitoes with the prior week's estimate. (Forty samples had less than 300 mosquitoes.) Supplementary Fig. 1 shows the weekly variability among years. Supplementary Table1 presents the annual number of weeks sampled each year, the total number of trap locations used within a year, the peak number of estimated infectious mosquitoes per 1,000 mosquitoes, the week that the peak number of infectious mosquitoes occurred, the number of *Culex* pools assayed for WNV, and the number of WNV positive *Culex* pools, as well as the number of reported human cases of WNV during 2001-2014.

Maximum Likelihood Estimation. It is assumed that if a pool tests positive at least one mosquito is WNV positive whereas a negative result indicates all mosquitoes are WNV negative. The maximum likelihood estimation (MLE) is considered the most appropriate estimate of infection rate when either pool size varies or infection levels are high[43]. County average mosquito infection rates were estimated using MLE and a binomial distribution, and all pooled samples for the county over a given week. Specifically, the log-likelihood equation for data $\boldsymbol{x}=(x_1, x_2, \ldots x_M)$ is:

$$l(p;x) = l(p) = \sum_{i=0}^{M} x_i log[1-(1-p)^{m_i}] + log(1-p)\sum_{i=0}^{M} m_i(n_i - x_i) \quad \textbf{[S6]}$$

where x_i is the number of positive samples for a given pool size, m_i is the distinct number

of mosquitoes sampled in a pool, n_i is the number of times the distinct pool size was sampled, and M is the number of distinct pool sizes. The solution to *p,* the maximum likelihood estimate of equation S6, was obtained using the Newton-Raphson method to iteratively compute successive values of *p* until convergence. The observational error variance (OEV) was then defined as the square of the standard error of the actual observed weekly mosquito infection rates from 2001 to 2014. If the OEV was less than 15, we set the OEV to 15. For more details on the MLE method to calculate a point estimate see Biggerstaff[29].

Description of EAKF. The ensemble adjustment Kalman filter (EAKF) is a data assimilation technique designed to estimate the true state of a system given both observations and model simulations of that state[22]. This algorithm has previously been used in conjunction with a variety of compartmental epidemiological models and infectious disease data to simulate diseases such as influenza and Ebola[17-20].

Kalman filters, in general, assume that at a given time *t* there is a mapping relation between the system state ($\boldsymbol{z_t}$) and observation space ($\boldsymbol{y_t}$). Given this relationship, Bayes' rule provides an updated estimate of the system state at time *t*, using the current observation, $\mathbf{y_t}$, and all prior observations, $\boldsymbol{y_{t-1}}$. The EAKF algorithm, specifically, is a deterministic form of the Kalman filter that uses an ensemble of simulations to generate a distribution of model states. In the presence of observations with prescribed OEV, the EAKF adjusts the ensemble of model-simulated state variables toward the target true state. Unobserved state variables and parameters are then adjusted as well using cross ensemble co-variability. When observational error is not correlated, the filter can be applied to multiple, simultaneously observed variables sequentially. For further details on how the EAKF adjusts the ensemble prior such that the new moments match the target moments of the posterior predicted by Bayes' theorem see Anderson[22].

Description of Combined Model-EAKF System. A 300- member ensemble simulation of the SIR compartmental model (Equations S1-S5) was run in conjunction with the Suffolk County infectious mosquito and human WNV case data and the EAKF. The filtering framework contains the modeled state space composed of the five disease state variables and four parameters $\boldsymbol{z_t}$=($S_M, I_M, S_B, I_B, I_H, \mu, \beta, \delta_B,$ and η) and the weekly observations of mosquito WNV infection rates and human WNV cases, $\boldsymbol{y_t}$=(I_M and I_H).

The observations were mapped directly to the state space variables using 2 simple scaling factors. The model assumes the mosquito population is constant (i.e. birth equals death); consequently, the scaling of observed mosquito WNV infection rates simply involved multiplying that observation by the model mosquito population. The scaling factor that relates observed human cases to modeled human cases is assumed to be one and is subsumed in the parameter representing the probability of transmission to humans from mosquitoes, η, for Suffolk County. Each week, in addition to updating the observed state variables, the EAKF algorithm also adjusts the unobserved state variables and model parameters using cross ensemble co-variability. The model is then integrated to the next observation using the updated (posterior) state variables and model parameters and the process is repeated. Over time this weekly, recursive adjustment using observed infected mosquitoes and human WNV cases optimizes the model state variables and parameters so that the ensemble model simulation better mimics local outbreak dynamics.

Development of the Beta term. Recent studies have used model-inference systems, such as the model-EAKF system described here, to provide estimates of critical epidemical parameters in both space and time[17,44]. Here, we used our combined model-EAKF system to simulate each WNV outbreak season in Suffolk County from 2001 to 2014 and provide posterior estimates of the model state variables and parameters. These estimates revealed that the contact rate, or probability of transmission between birds and mosquitoes, β, the recovery time for infected birds, δ_B, and the mosquito birth and death rate, μ_M, exhibited strong co-variability to estimate infected mosquitoes over time. Typically, around the onset of an outbreak β and δ_B would be high and μ_M low allowing the virus to amplify in the mosquitoes and birds, and the epidemic would decline as μ_M increased and β and δ_B decreased. The increased mosquito death rate, μ_M, removed infected mosquitoes from the system, and the reduced bird infectious period along with the reduced contact rate prevented future susceptible mosquitoes from becoming infected.

This reduction of contact rate between mosquitoes and birds during a biting season matches field observations. Studies in Florida, Colorado, California and Connecticut have shown that a number of *Culex* mosquito species transition from preferential feeding on birds to mammals over the course of a season[23-26]. This change in mosquito feeding preference is consistent with the simulated decrease in the posterior estimates of β at each observation point from 2001 to 2014 (Supplementary Fig. 2). This shift of β, which in form follows a generalized logistic function, may stem from avian dispersal or mosquito biting preference shifts in Suffolk County. It is an important dynamical feature of the system, as prediction of future mosquito infection levels requires prediction of this change of β. We therefore incorporated this potential for a shift of β explicitly in the SIR model. Specifically, we replaced β with the generalized logistic equation:

$$\boldsymbol{\beta(t) = A + \frac{K-A}{1+e^{\left(-r(t-t_0)\right)}}} \qquad \textbf{[S7]}$$

where A is the lower asymptote, K is the upper asymptote, r is the growth rate, and t_0 is the inflection point. By imposing this form within the model, we remove the parameter β and add the 4 parameters, K, A, r and t_0. Successful predictions of future shifts in feeding preference and vector-avian host contact require sufficient optimization of these 4 new parameters in Equation S7. With Equation S7, the model (Equations 1-5) consists of five disease state variables and seven parameters $\boldsymbol{z_t}$=(S_M, I_M, S_B, I_B, I_H, A, K, r, t_0, μ, δ_B, and η).

Generation of Synthetic Truth and Observation. To validate EAKF optimization of the WNV model, we generated a synthetic, model-simulated WNV outbreak. This synthetic outbreak, defined as the "truth", was generated by free simulation of the model (Equations 1-6) for a single WNV season (June 1st to October 31st). We initiated this simulation with all mosquitoes, birds and humans susceptible and model parameters μ=0.065, A=0.02, K=0.08, r=-0.08, t_0=220, δ=5.4, and η=0.0045. The simulation was then seeded with infected mosquitoes, α, during integration until the middle of July at a rate of 1 in 500,000. The parameter combination was chosen to produce a good representation of the mean weekly estimate of infectious mosquitoes and reported human

cases of WNV for Suffolk County from 2001 to 2014. Sampling of the simulated truth every 7 days then formed a time series of the "true" number of infected mosquitoes and reported human cases of WNV. Synthetic observations of infected mosquitoes were then generated by adding normally distributed random observational error (mean 0 and standard deviation equal to the standard error of the actual observed weekly mosquito infection rates from 2001 to 2014) to the truth. Synthetic observations of humans infected with WNV were generated directly from the time series analysis using a Poisson random number generator. These synthetic error-laden observational records of infectious mosquitoes and reported human WNV cases were then used for assimilation in the combined model-EAKF system.

Application of Synthetic Observations to the Model-Inference System. The synthetic observations of weekly-infected mosquitoes and human WNV cases, along with their defined OEV, were used to determine whether the model-EAKF system could appropriately estimate unobserved state variables and parameters. For these optimization tests, we used a 300-member ensemble of model simulations[32]. Each ensemble member was initialized with a constant total population: $S_{m(0)}$ =4,000- $I_{m(0)}$, $I_{m(0)}=0$, $S_{B(0)}$=500- $I_{B(0)}$, $I_{B(0)}=0$ and $I_{H(0)}$=0; initial model parameters were randomly selected from a uniform distribution: μ=U(0.05,0.09), A= U(0.01,0.04), K= U(0.06,0.13), r= U(-0.12, -0.02), t_0= U(206,227), δ= U(3.8,6.0), and η= U(0,0.005). The model was run using a daily time step and the EAKF was used to assimilate the weekly synthetic observations of infected mosquitoes and human WNV cases. We evaluated 10 different observational data sets and each observational data set was simulated 100 times to account for the random selection of initial state variables and parameters.

Overall, the ensemble posterior mean state variable and parameter estimates were well constrained (Supplementary Figs. 3-6). The two observed state variables, I_M, infectious mosquitoes, and I_H, infectious humans, were both well captured in the combined model-EAKF framework (Supplementary Fig. 3); however, when only one observational data stream was assimilated model constraint was degraded (not shown). For example, when only infected mosquitoes, I_M, were assimilated, model estimates of the number of reported human WNV cases were less well constrained. In contrast, when only I_H was assimilated, due to weaker optimization of η, the parameter that scales the proportion of infected mosquitoes to the number of human cases, estimates of true mosquito infection rates were weaker. Overall, the combined model-EAKF framework performed best when both data streams were assimilated.

The unobserved state variables, S_M, susceptible mosquitoes, S_B, susceptible birds, and I_B, infectious birds, were also well captured in the ensemble posterior mean (Supplementary Fig. 4). In addition, the epidemiologically significant parameters within the model that help define the number of human cases over a season, along with peak timing and magnitude of infectious mosquitoes during an outbreak were also appropriately estimated by the EAKF. Specifically, parameters η, A, and r adjusted toward the truth in response to assimilation of I_M and I_H observations. Parameters A, K, r and t_0 determine the contact rate between birds and mosquitoes, $\beta(t)$ (Equation 6 or Equation S7). The EAKF constrains $\beta(t)$ very well (Supplementary Fig. 5), even though only two parameters, r and A, are adjusted toward the truth (Supplementary Fig. 6). Parameter K, the upper asymptote of $\beta(t)$, is consistently high in the early stages or the amplification period of an outbreak due to limited information pertaining to the outbreak.

As time progresses and more information is observed about the outbreak, $\beta(t)$ is adjusted to counter the high upper asymptote by making the infection point, t_0, earlier than the truth. The early t_0 brings $\beta(t)$ closer to the truth and by this point in the simulations, the EAKF has constrained the parameter r so that $\beta(t)$ closely follows the truth as it transitions from the upper asymptote to the lower asymptote. The lower asymptote, A, is adjusted closer to the truth by the end of the simulation, but still remains slightly high of the target, yielding a slightly high estimate of $\beta(t)$. To counteract this bias, the mosquito birth and death rate, $\mu_{M,}$ remained higher than the truth and the duration of bird infection, δ_B, was slightly lower than the truth (Supplementary Figs. 5 and 6). All four of these parameters exhibited strong covariability across the ensemble. While this covariability inhibits precise EAKF estimation of all true parameter values, functional estimates are generated in which estimation biases appear to compensate for one another. The rate of human spillover, η, was adjusted due to assimilation of I_H and converges toward the truth as more observations are brought to bear.

Forecast Procedure. Weekly ensemble forecasts of future mosquito WNV infection rates and human WNV cases are generated following ensemble optimization up to the point of forecast. Optimization, or training, begins following initiation of an ensemble simulation at the start of a particular season and is critical for developing an appropriate set of initial conditions for the actual forecast. In practice, observations are assimilated iteratively from the start of the simulation up to the point of forecast. The assimilation further optimizes the ensemble with each weekly observation of mosquito infection rates and human cases. By repeatedly adjusting the ensemble of simulations, model state variables and parameters are better aligned to represent the observed outbreak as it has thus far manifest. The expectation is that if the ensemble of simulations better represents the outbreak as observed up to the present, it will generate a more accurate forecast of future infection rates and human cases. Those forecasts are generated using the latest posterior estimates of the model state variables and parameters by integrating the compartmental model (Equations 1-6) through time until the end of the outbreak. This process is repeated on a weekly basis until the end of the outbreak, i.e. each successive forecast has one more week of optimization.

Retrospective Forecast. The forecast procedure was used to generate retrospective forecasts of WNV outbreaks from 2001 to 2014. An ensemble compartmental-model was initiated with a 300-member ensemble for each outbreak season (June to November). Each ensemble member was initialized with constant total population: $S_{m(0)}$ =4,000- $I_{m(0)}$, $I_{m(0)}=0$ $S_{B(0)}$=500- $I_{B(0)}$, $I_{B(0)}=0$ and $I_{H(0)}$=0; and model parameters were randomly selected from uniform distributions: μ=U(0.05,0.09), A= U(0.001,0.015), K= U(0.06,0.1), r= U(-0.2, -0.05), δ= U(3.8,6.0), η= U(0,0.004), and t_0 was 5 to 10 weeks after the appearance of the first infectious pool of mosquitoes. The simulation was seeded with infected mosquitoes, α, during integration until day 200 at a rate of 1 in 500,000. For each outbreak, model optimization began four weeks prior to observation of the first infected mosquito pool and weekly forecasts were generated once the first pool of infected mosquitoes was detected, typically during early July. Each ensemble forecast was repeated 10 times with different randomly selected initial conditions.

Analysis of Retrospective Forecasts. The quality of the retrospective seasonal forecasts was analyzed through comparison to observations to determine how well each ensemble forecast estimated the peak timing and peak magnitude of infectious mosquitoes, seasonal total infectious mosquitoes, and the number of human cases for the season. For all 4 metrics we compared the ensemble mean trajectory with observed outcomes. Forecasts were considered accurate if: 1) it peaked within ±1 week of the observed peak of infectious mosquitoes; 2) the maximum mosquito infection rate was within ±25% of the observed peak infection rate; 3) the total number of infectious mosquitoes over the entire season was within ±25% of the observed; and 4) the total number of human cases over the entire season was within ±25% or ±1 case of the total number of reported cases, whichever was larger. As an additional analysis, forecasts were grouped by prediction lead, i.e. how many weeks in the future or past the outbreak peak is predicted to occur or to have occurred. All forecasts with the same lead were grouped and the fraction of accurate forecasts was quantified.

Cook County Forecast

Study Area.
WNV first arrived in Cook County in 2002 and since has generated 1,252 reported human cases of WNV, more than any other county in the U.S., prior to 2015[30]. Cook County is the second most populous county in the United States. It is made up of the city of Chicago and 30 suburban townships with a total population of approximately 5.2 million people. The county borders the southwest portion of lake Michigan with a land area of 945 square miles with natural landscape and land use patterns throughout the county that provide ample breading sites for *Culex spp*. mosquitoes, the primary vectors of WNV.

Observed Human Cases. Weekly human cases of WNV in Cook County Illinois were obtained from ArboNET, the national arboviral surveillance system, from 2007 to 2014[30].

Observed Infectious Mosquitoes. Mosquito surveillance in Cook County Illinois was conducted weekly from the middle of May to the middle of October. Data were obtained from the Chicago mosquito monitoring program and Desplaines Valley Mosquito Abatement District (two of the five mosquito abatement districts in Cook County). Total traps within a season ranged from 98 to 136 depending on the year, and the number of traps set each week varied from 6 to 131 traps. For abroviral analysis, pools from the Chicago mosquito monitoring program were submitted to the Chicago Department of Public Health environmental laboratory for WNV testing. Pools collected by the Desplaines Valley Mosquito Abatement District were submitted to Illinois Natural History Survey for WNV testing. In both instances, PCR testing of pooled mosquitoes was used to detect the presence of WNV. We combined the results from all mosquito pools tested in a week and used a maximum likelihood approach to estimate the total weekly proportion of positive mosquitoes[29].

Cook County Forecast Procedure. Forecasting was carried out as for Suffolk County. Initial conditions for forecasting were the same with the exception of η, the scalar that accounts for spillover contact and the probability of transmission from mosquitoes to humans. Initial η is U(0,0.0136), since the population of cook county is 3.4 times that of Suffolk county.

Forecast Results. Supplementary Fig. 13 shows forecast accuracy for Cook County during 2007-2014. Forecasts of peak timing were >55% accurate with six-week lead. Forecasts of peak infectious mosquito number were >40% accurate when the predicted with a 0-week lead and >69% past the peak. For the total number of infected mosquitoes 45% of forecasts were accurate one week prior to the predicted peak, 50% were accurate at the predicted peak and 87% were accurate one week past the peak. At 0, 1 and 2 weeks past peak predicted mosquito infection rates, forecasts of total human WNV cases were accurate 40%, 74% and 69% of the time, respectively. On average, only 18% of human cases had been reported at the peak, 34% of human cases had been reported one week past the peak and 46% had been reported 2 weeks past the peak. Human cases were accurately forecast 5 to 11 weeks prior to the end of the outbreak (Supplementary Fig. 14).

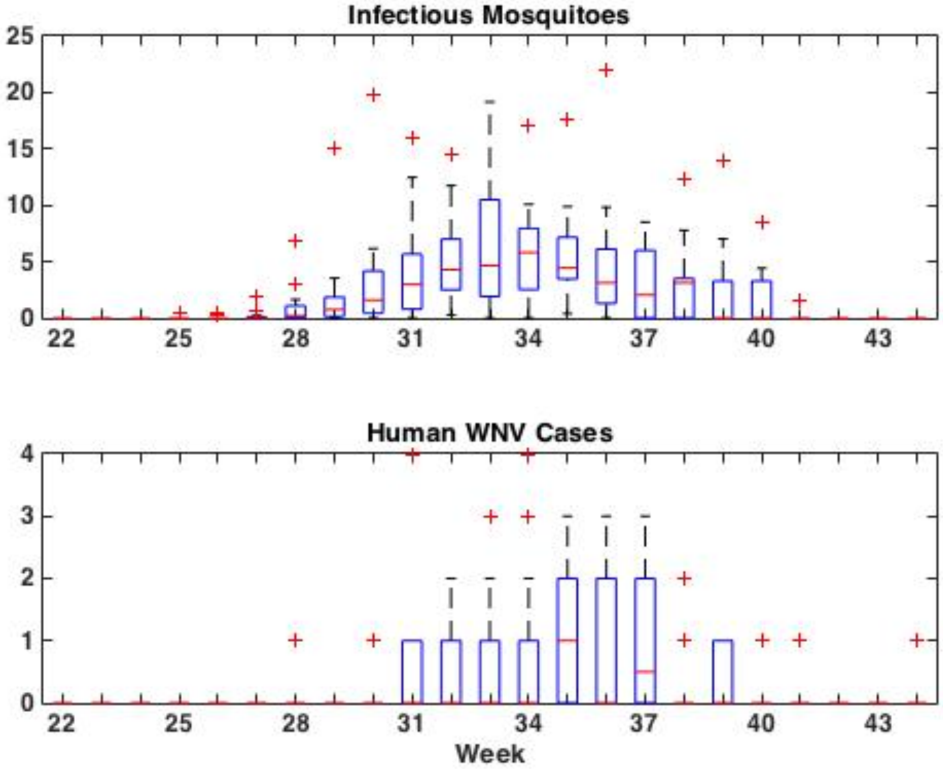

Supplementary Figure 1. Time series of the number of infected mosquitoes per 1,000 tested and the number of reported human WNV cases from 2001 to 2014. The box and whiskers shows show the median (red horizontal line), 25th and 75th percentiles (box boundaries), the whiskers mark the highest and lowest values within 1.5 times the inter quartile range of the box boundaries and outliers (red +). Human WNV cases were lag correlated to the number of infected mosquitoes per 1,000 tested for the prior week, ($r = 0.56$, $p < 0.001$). This coincides with the incubation period for clinical illness, which generally ranges from 2 and 14 days[6].

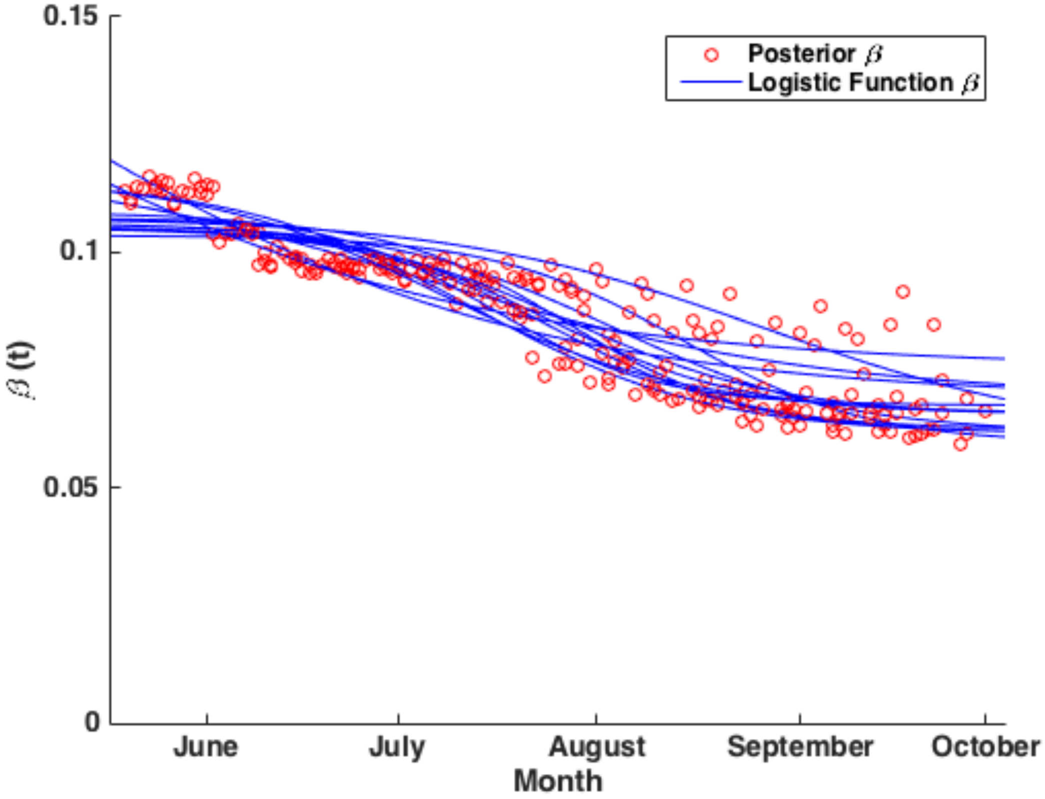

Supplementary Figure 2. The red circles are the ensemble mean posteriors estimates of β from simulation with the SIR-EAKF system for each season from 2001 to 2014. The blue lines show logistic functions fit to those mean ensemble posterior estimates of β for each outbreak from 2001 to 2014, per the methods of Cavallini[45].

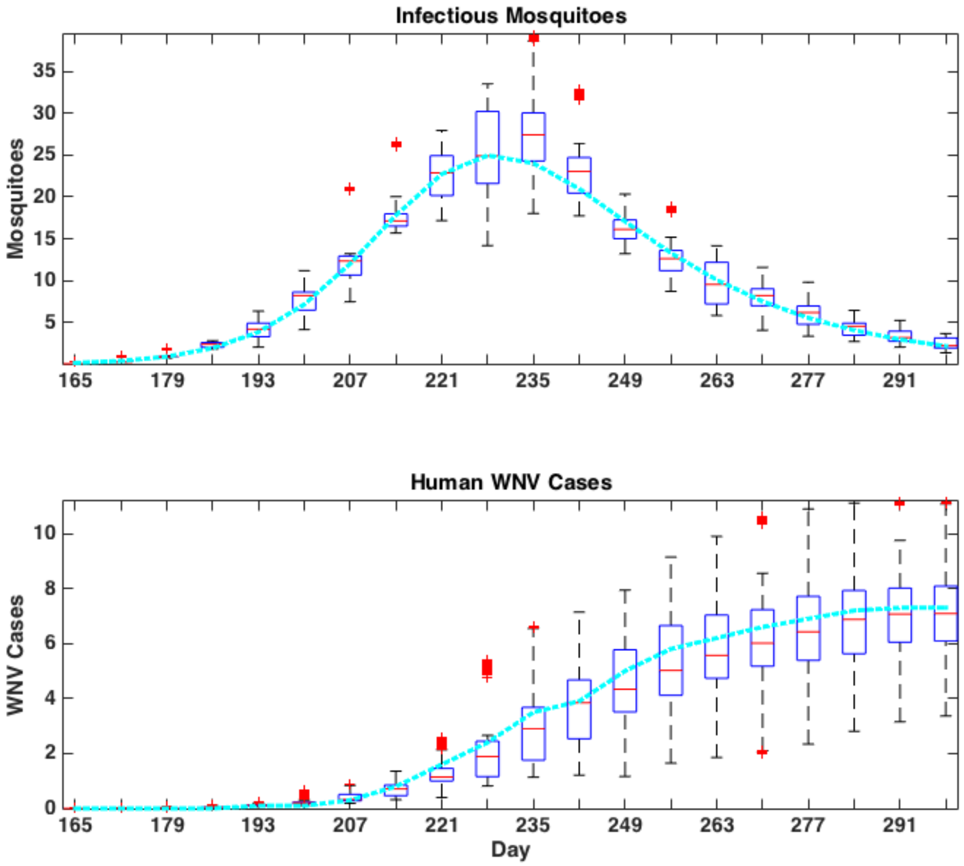

Supplementary Figure 3. Time series results of the posterior ensemble mean for the observed state space, I_M and I_H, generated using the compartmental model-EAKF framework, and truth, cyan colored dotted line. The 1,000 simulations are represented in the box and whiskers, which show the median (red horizontal line), 25th and 75th percentiles (box boundaries), the whiskers mark the highest and lowest values within 1.5 times the inter quartile range of the box boundaries and outliers (red +).

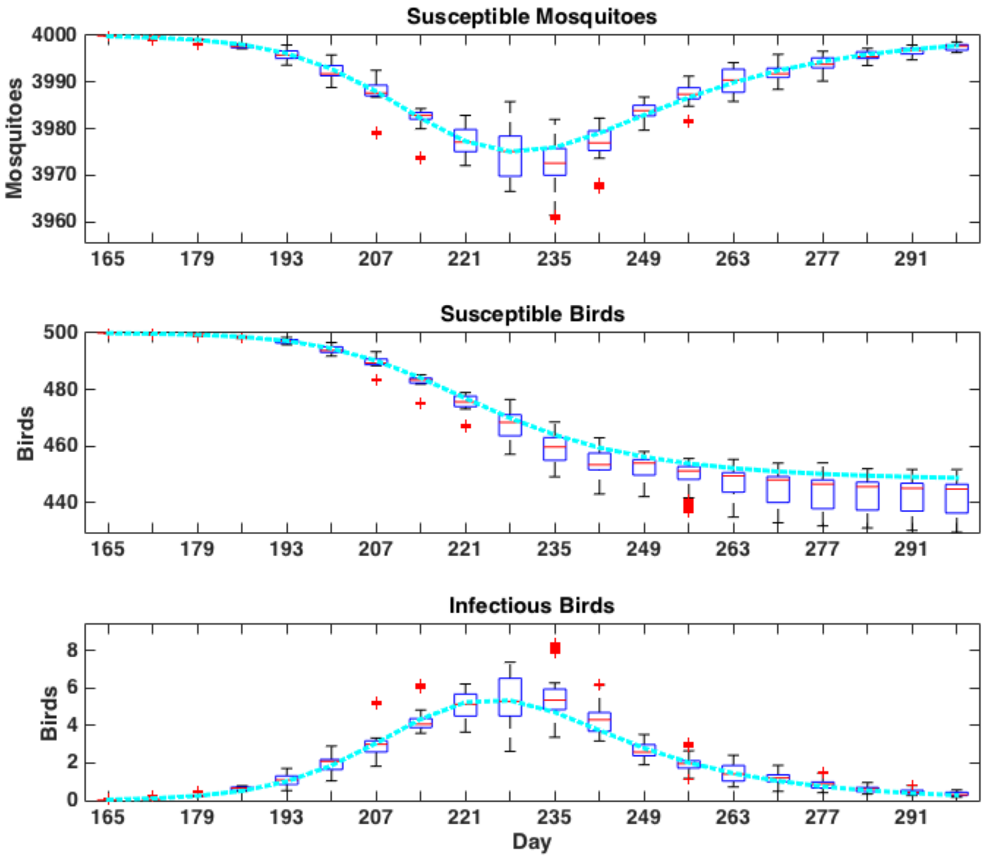

Supplementary Figure 4. Time series results of the posterior ensemble mean for the unobserved state space, S_M, S_B and I_B, generated using the compartmental model-EAKF framework, and truth, cyan colored dotted line. The box and whiskers shows show the median (red horizontal line), 25th and 75th percentiles (box boundaries), the whiskers mark the highest and lowest values within 1.5 times the inter quartile range of the box boundaries and outliers (red +).

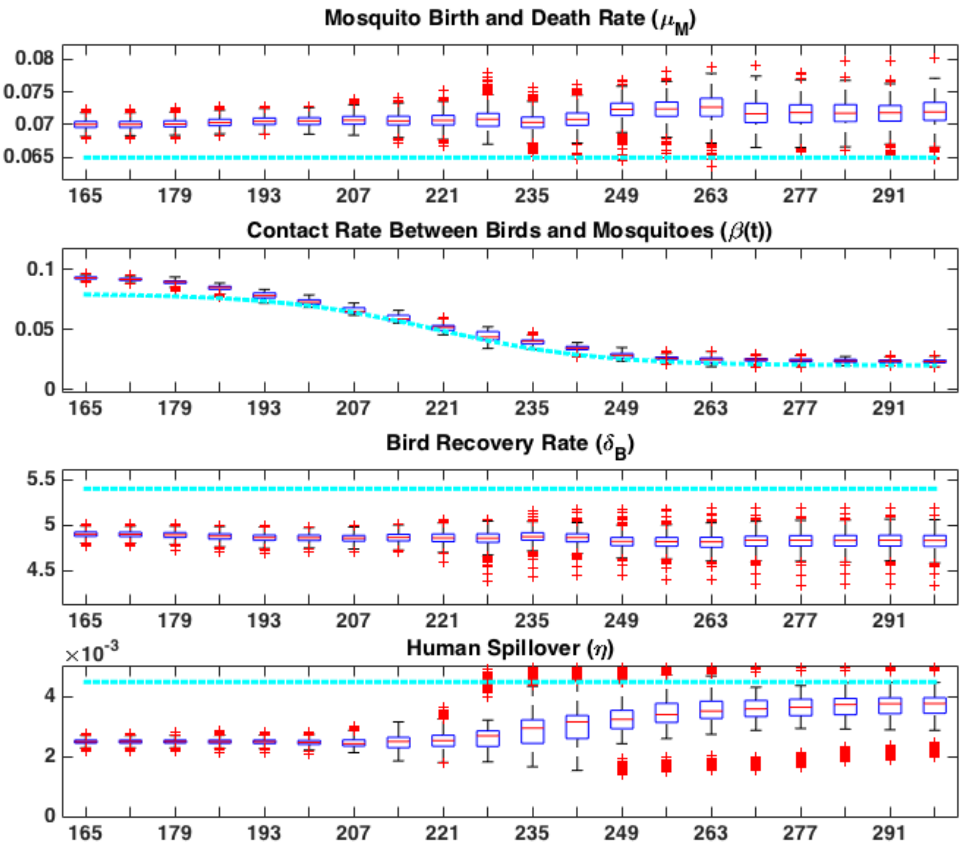

Supplementary Figure 5. Time series results of the posterior ensemble mean for parameters μ_M, $\beta(t)$, δ_B, and η, generated using the compartmental model-EAKF framework, and truth, cyan colored dotted line. The box and whiskers shows show the median (red horizontal line), 25th and 75th percentiles (box boundaries), the whiskers mark the highest and lowest values within 1.5 times the inter quartile range of the box boundaries and outliers (red +).

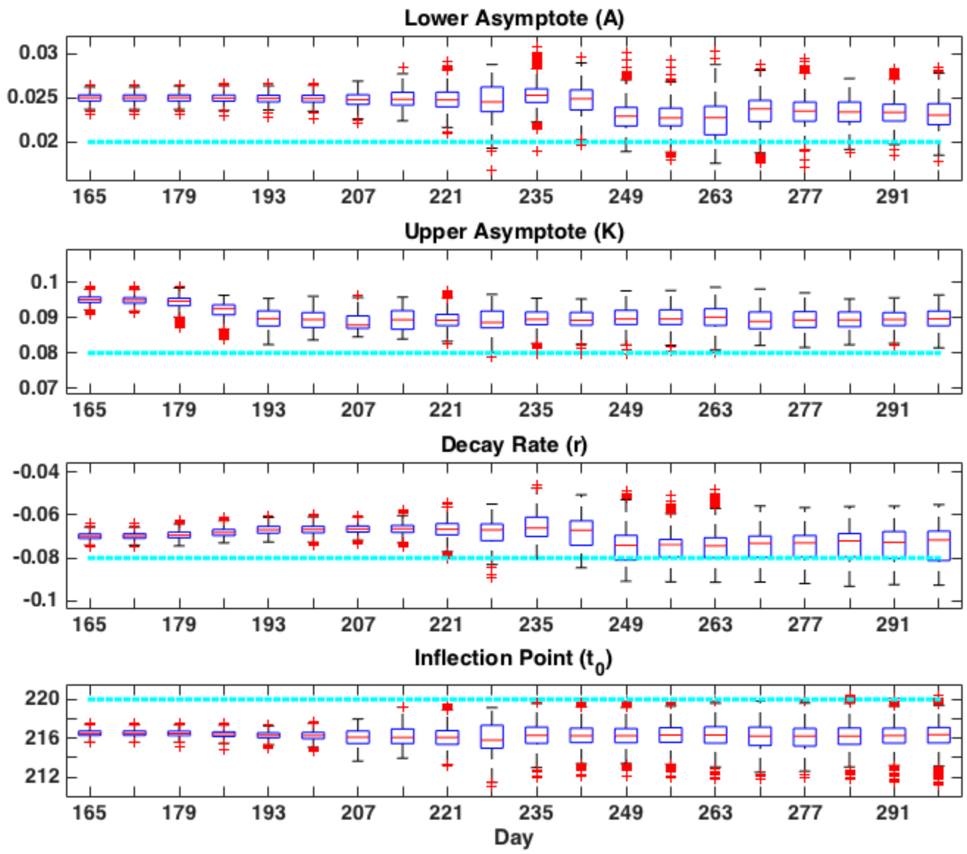

Supplementary Figure 6. Time series results of the posterior ensemble mean for parameters *A, k, r,* and t_0, generated using the compartmental model-EAKF framework, and truth, cyan colored dotted line. The box and whiskers shows show the median (red horizontal line), 25th and 75th percentiles (box boundaries), the whiskers mark the highest and lowest values within 1.5 times the inter quartile range of the box boundaries and outliers (red +).

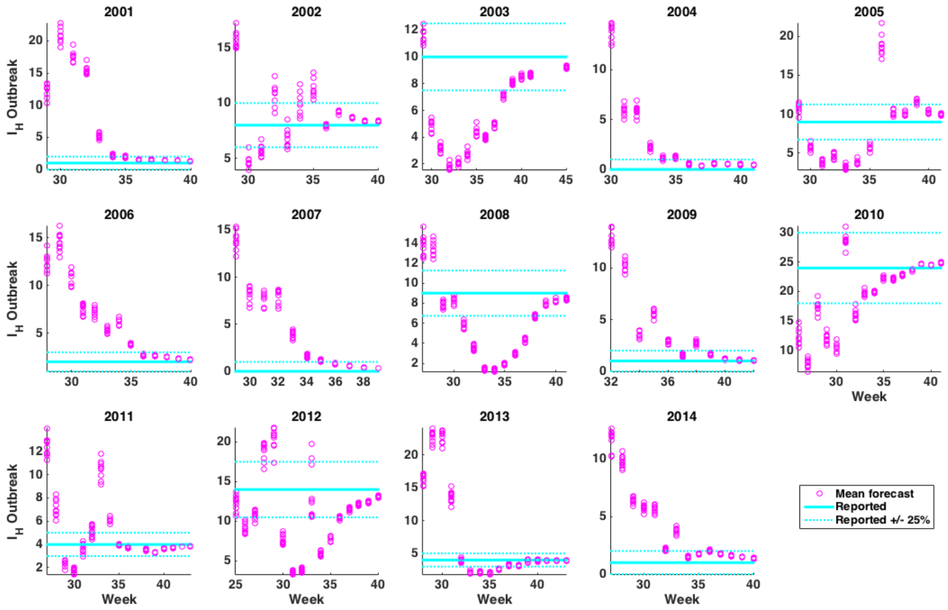

Supplementary Figure 7. Forecast accuracy of ten model-EAKF system predictions of human WNV cases for each season. The purple circles give weekly ensemble mean predictions of human WNV cases for each season. The solid cyan line depicts total observed human cases for each season; the dotted cyan line defines values within ±25% or ±1 case of the total number of reported cases. Forecasts were accurate within ±25% or ±1 case by week 35 in 6 of 14 years.

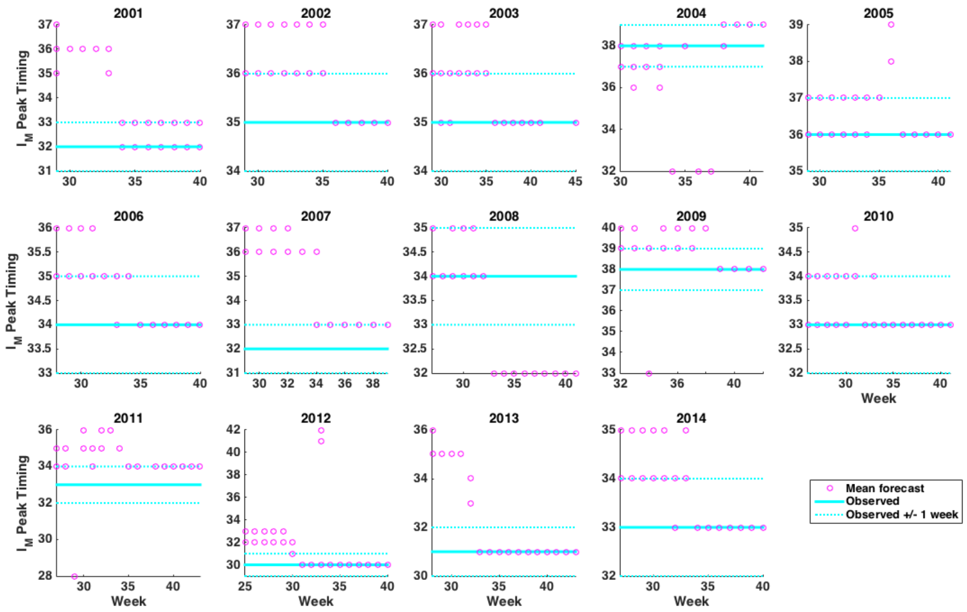

Supplementary Figure 8. Forecast accuracy of ten model-EAKF system predictions for the week of peak mosquito infection for each season. The purple circles give weekly ensemble mean predictions of peak timing. The solid cyan line depicts the week of the observed peak for each season; the dotted cyan line defines values within ±1 week of the observed. Forecasts were accurate within ±1 week by week 35 in 10 of 14 years.

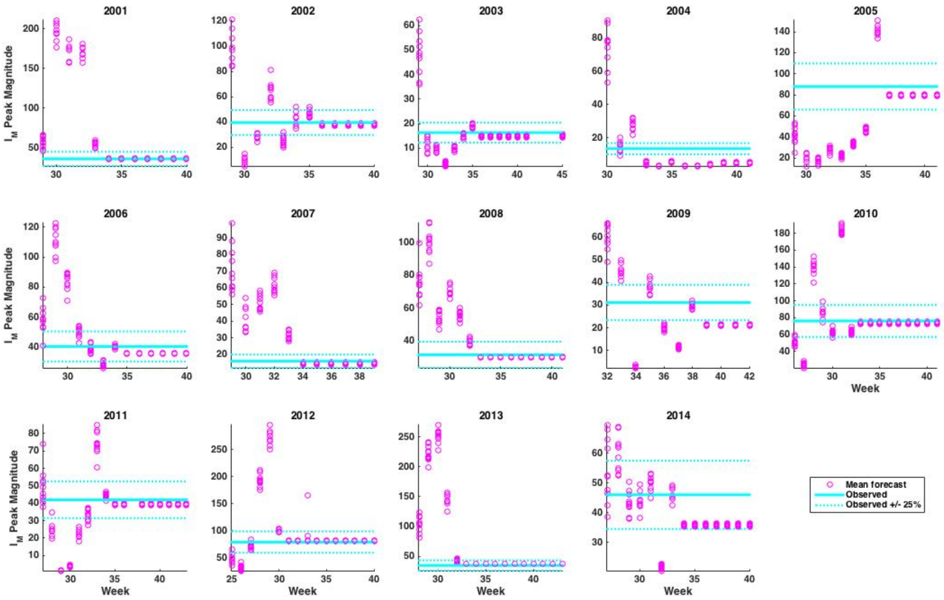

Supplementary Figure 9. Forecast accuracy of ten model-EAKF system predictions for the magnitude of peak mosquito infection for each season. The purple circles give weekly ensemble mean predictions. The solid cyan line depicts the observed peak magnitude for each season; the dotted cyan line defines values within ±25% of the observed. Forecasts were accurate within ±25% by week 35 in 11 of 14 years.

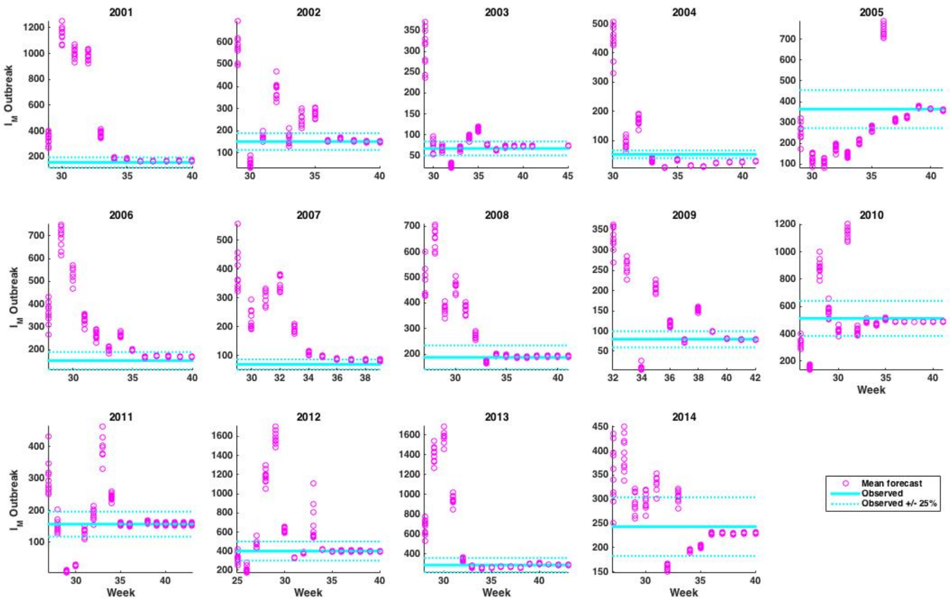

Supplementary Figure 10. Forecast accuracy of ten model-EAKF system predictions for the total number of infected mosquitoes for each season. The purple circles give weekly ensemble mean predictions. The solid cyan line depicts the observed total number of infected mosquitoes for each season; the dotted cyan line defines values within ±25% of the observed. Forecasts were accurate within ±25% by week 35 in 9 of 14 years.

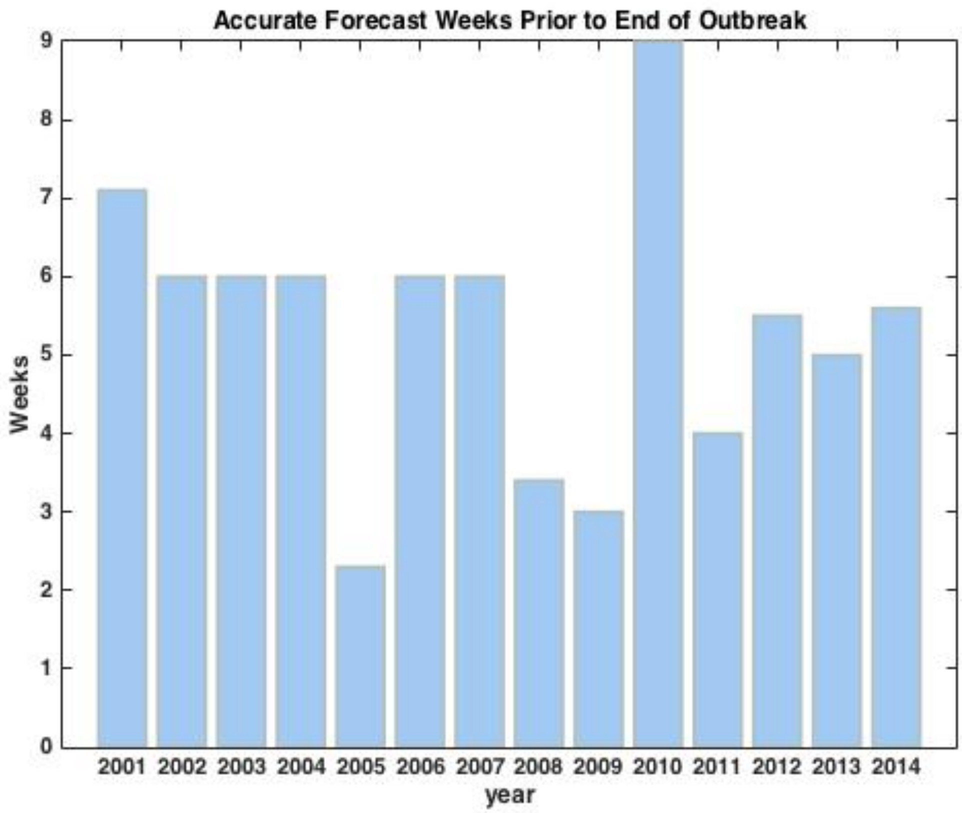

Supplementary Figure 11. The number of weeks of accurate forecast of total human WN cases prior to week 42, the typical last week of the outbreak. For 2003 the outbreak ended in week 45, so weeks prior to week 45 are presented. Each bar is the average of 10 ensemble forecasts (see Methods), hence the values are continuous not integer.

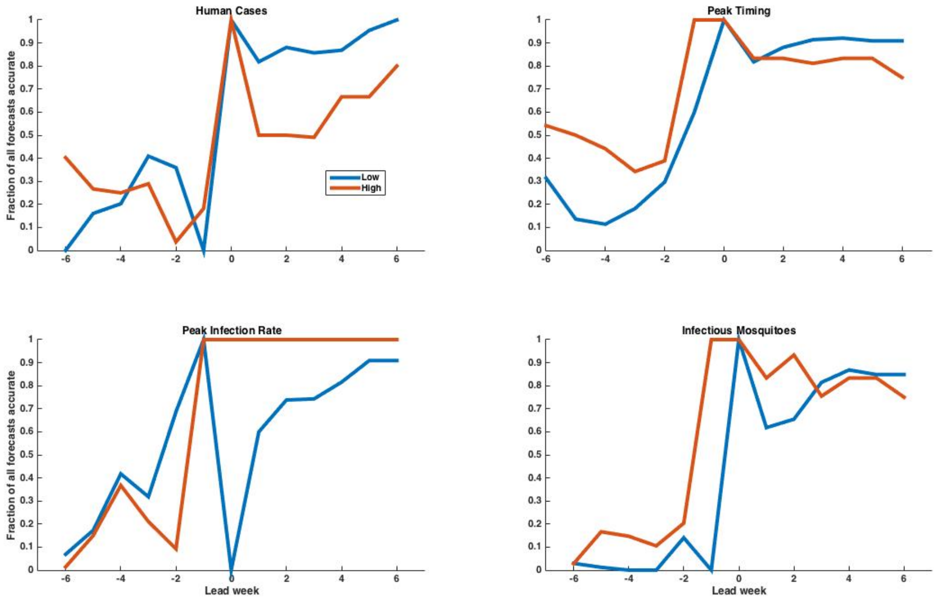

Supplementary Figure 12. 2001-2014 retrospective forecasts divided into two groups based on the number of human cases reported each year. The low group (2001, 2004, 2006, 2007, 2009, 2011, 2013 and 2014) is years with 4 or fewer reported human WNV cases (blue); the high group (2002, 2003, 2005, 2008, 2010 and 2012) is years with 8 or more reported human cases (orange). Shown are the fraction of accurate forecasts as a function of lead week. A forecast was deemed accurate if: 1) peak timing was within ±1 week of the observed peak of infectious mosquitoes; 2) peak infection rate was within ±25% of the observed peak infection rate; 3) total infectious mosquitoes were within ±25% of the observed; and 4) human WNV cases were within ±25% or ±1 case of the total number of reported cases, whichever was larger.

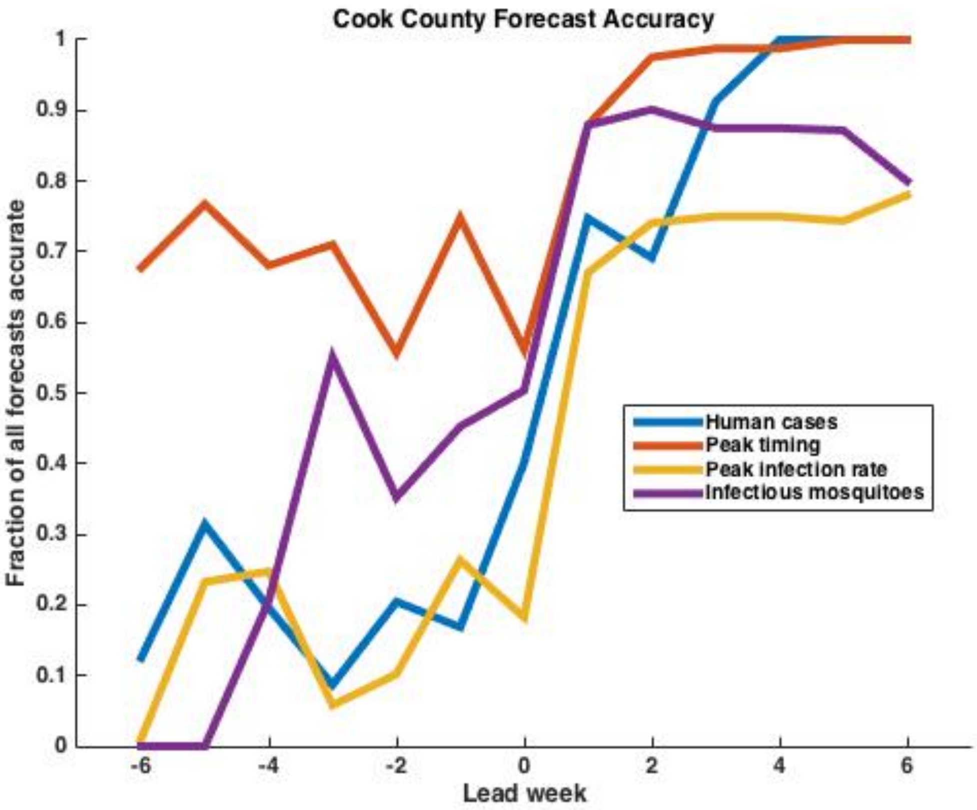

Supplementary Figure 13. Results for 2007-2014 retrospective forecasts. Shown are the fraction of forecasts accurate as a function of lead week for the metrics human WNV cases (blue), peak timing (week of peak mosquito infection rates, orange), peak infection rate (yellow), and total infectious mosquitoes (purple). A forecast was deemed accurate if: 1) peak timing was within ±1 week of the observed peak of infectious mosquitoes; 2) peak infection rate was within ±25% of the observed peak infection rate; 3) total infectious mosquitoes were within ±25% of the observed; and 4) human WNV cases were within ±25% or ±1 case of the total number of reported cases, whichever was larger. Note that for all metrics lead week is shown with respect to the week of peak mosquito infection.

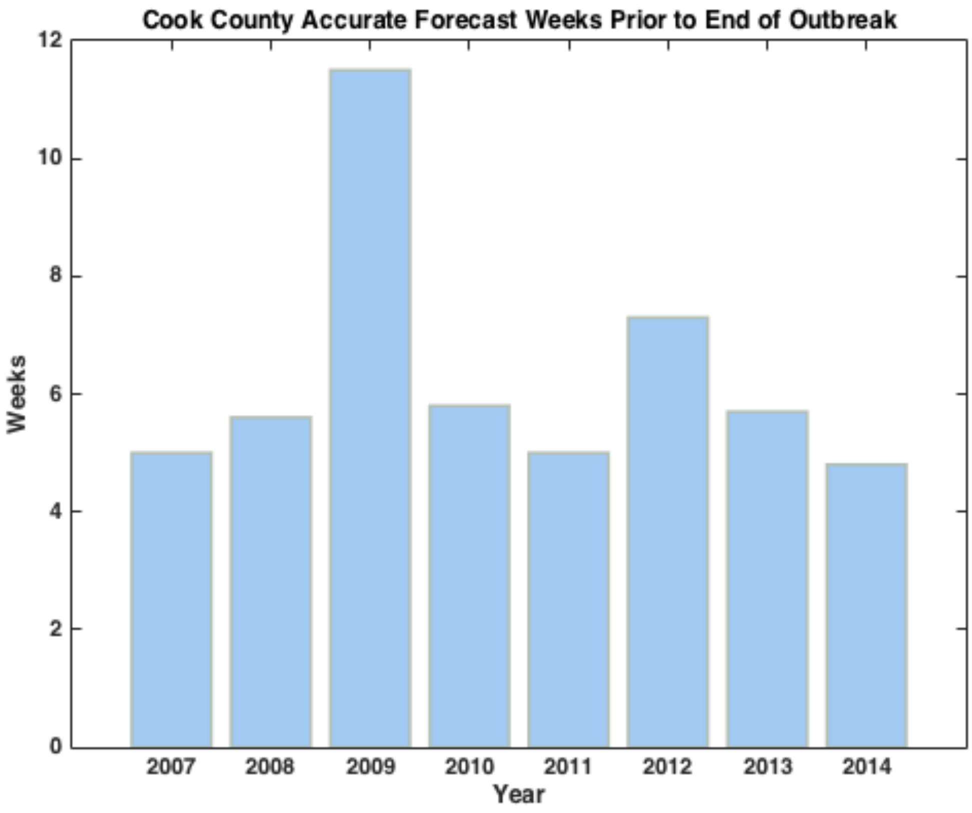

Supplementary Figure 14. The number of weeks of accurate forecast of total human WN cases prior to week 42, the typical last week of the outbreak. Each bar is the average of 10 ensemble forecasts (see Methods), hence the values are continuous not integer.

Supplementary Table 1. Overview of Human WNV Cases, Mosquito Infection Rates, and Mosquito Data in Suffolk County 2001-2014

Year	Human cases	Weeks sampled	No. of trap locations	Positive weeks	Peak infection rate*	Peak timing^	No. of *Culex* pools tested	No. of WNV-positive *Culex* pools
2001	1	18	75	10	9	8/11/01	721	48
2002	8	18	101	9	10	8/31/02	762	30
2003	10	20	88	8	4	8/30/03	1088	33
2004	0	19	58	5	4	10/2/04	613	7
2005	9	19	104	12	22	9/3/05	1051	69
2006	2	19	62	10	10	8/26/06	858	54
2007	0	18	47	8	4	8/11/07	432	12
2008	9	19	95	12	8	8/23/08	644	41
2009	1	18	50	6	8	9/19/09	775	15
2010	24	19	98	14	20	8/14/10	1473	285
2011	4	20	71	11	10	8/13/11	1338	79
2012	14	17	54	12	20	7/28/12	912	204
2013	4	21	58	14	9	8/3/13	1332	178
2014	1	18	51	14	12	8/16/14	1347	186

*Per 1,000 mosquitoes estimated using the MLE

^ Last day of the week

Supplementary References:

36 Andreadis, T. G., Anderson, J. F., Vossbrinck, C. R. & Main, A. J. Epidemiology of West Nile virus in Connecticut: a five-year analysis of mosquito data 1999-2003. *Vector-Borne & Zoonotic Diseases* **4**, 360-378 (2004).

37 Kulasekera, V. L. *et al.* West Nile virus infection in mosquitoes, birds, horses, and humans, Staten Island, New York, 2000. *Emerging Infectious Diseases* **7**, 722 (2001).

38 Hamer, G. L. *et al.* Culex pipiens (Diptera: Culicidae): a bridge vector of West Nile virus to humans. *Journal of Medical Entomology* **45**, 125-128 (2008).

39 Kilpatrick, A. M. *et al.* West Nile virus risk assessment and the bridge vector paradigm. *Emerg Infect Dis* **11**, 425-429 (2005).

40 Crabtree, M., Savage, H. & Miller, B. Development of a species-diagnostic polymerase chain reaction assay for the identification of Culex vectors of St. Louis encephalitis virus based on interspecies sequence variation in ribosomal DNA spacers. *The American Journal of Tropical Medicine and Hygiene* **53**, 105-109 (1995).

41 Bernard, K. A. & Kramer, L. D. West Nile virus activity in the United States, 2001. *Viral Immunology* **14**, 319-338 (2001).

42 Lukacik, G. *et al.* West Nile virus surveillance in mosquitoes in New York State, 2000-2004. *Journal of the American Mosquito Control Association* **22**, 264-271 (2006).

43 Gu, W., Lampman, R. & Novak, R. Assessment of arbovirus vector infection rates using variable size pooling. *Medical and Veterinary Entomology* **18**, 200-204 (2004).

44 Bretó, C., He, D., Ionides, E. L. & King, A. A. Time series analysis via mechanistic models. *The Annals of Applied Statistics*, 319-348 (2009).

45 Cavallini, F. Fitting a logistic curve to data. *College Mathematics Journal* **24**, 247-253 (1993).
